# Secreted biofilm factors adversely affect cellular wound healing responses *in vitro*

**DOI:** 10.1038/srep13296

**Published:** 2015-08-17

**Authors:** Robert Jeffery Marano, Hilary Jane Wallace, Dulharie Wijeratne, Mark William Fear, Hui San Wong, Ryan O’Handley

**Affiliations:** 1 Ear Science Institute, Suite 1, Level 2, 1 Salvado Road, Subiaco, WA 6008, Australia; 2 Ear Sciences Centre, School of Surgery, The University of Western Australia, Crawley, Western Australia; 3 Burn Injury Research Unit, School of Surgery, The University of Western Australia, Western Australia, Australia; 4 Fiona Wood Foundation, Western Australia, Murdoch 6150, Australia; 5 School of Animal and Veterinary Sciences, The University of Adelaide, Roseworthy Campus, Adelaide, South Australia, Australia

## Abstract

Although most chronic wounds possess an underlying pathology, infectious agents also contribute. In many instances, pathogens exist as biofilms forming clusters surrounded by a secreted extracellular substance. We hypothesized that compounds secreted by biofilm bacteria may inhibit normal wound healing events including cell proliferation and migration. Conditioned media from two common bacterial species associated with chronic skin wounds and chronic tympanic membrane perforations, *Staphylococcus aureus* and *Pseudomonas aeruginosa*, were evaluated for their capacity to affect keratinocyte proliferation and migration. Additionally, proteomic analysis was performed to identify proteins within the biofilm conditioned media that may contribute to these observed effects. Biofilm conditioned media from both species inhibited proliferation in human tympanic membrane derived keratinocytes, whereas only biofilm conditioned media from *S. aureus* inhibited migration. Human epidermal keratinocytes were found to be more sensitive to the effects of the conditioned media resulting in high levels of cell death. Heat treatment and microfiltration suggested that *S. aureus* activity was due to a protein, while *P. aeruginosa* activity was more likely due to a small molecule. Proteomic analysis identified several proteins with putative links to delayed wound healing. These include alpha hemolysin, alcohol dehydrogenase, fructose-bisphosphate aldolase, lactate dehydrogenase and epidermal cell differentiation inhibitor.

The primary function of the skin is to serve as a barrier to the external environment. Wound healing of the skin is a complex process involving cellular proliferation, migration and tissue remodelling leading to reestablishment of its primary function. Failure of a wound to heal in a timely manner may lead to further complications, including septicemia, chronic pain, prolonged hospitalization, amputations, and mortality^1^. Chronic wounds in humans include wounds of the lower limb, due to an underlying pathology such as diabetes, pressure or venous hypertension, and chronic tympanic membrane perforations typically caused by chronic suppurative otitis media. Bacteria are present in chronic wounds and are proposed to play a role in delayed tissue repair^2^,^3^.

It is considered that the bacteria present in chronic wounds not only exist in a planktonic form, but are also characterized by polymicrobial colonies of bacteria existing in communities known as biofilms^4^. Biofilms consist of a cluster of bacteria adhering to a surface surrounded by an extracellular polymeric matrix^5^. The function of the biofilm is varied and includes protection from the environment, nutrient storage and aiding in cell-cell communication. Consequently, gene expression and phenotype of bacteria in biofilm mode is different to that when in free living or planktonic mode^6^,^7^. The presence of a biofilm in chronic wounds gives rise to several common features; they render the bacteria virtually impervious to antibiotic treatments^8^ and affected tissues remain in a persistent inflammatory state^1^. Additionally, it has been shown that the presence of a biofilm can delay wound healing^9^. However, the mechanisms of delayed wound healing due to biofilms are poorly understood.

We hypothesize that bioactive compounds secreted by bacteria whilst in biofilm mode inhibit key wound healing mechanisms including cell proliferation and migration. Therefore, the aims of this research were to grow isolates of bacteria in biofilm mode and planktonic mode and produce conditioned media. We have selected perhaps the two most common bacterial species related to chronic wound colonization or infection, *Pseudomonas aeruginosa* and *Staphylococcus aureus*. *In vitro* cell culture was then used to compare the effects of secreted biofilm and planktonic compounds on key wound healing events, cell proliferation and migration. For this purpose we utilized human derived primary keratinocytes from two distinct areas, the tympanic membrane and the normal epidermis of the upper arm. Subsequently, proteomic analysis was undertaken to identify potential protein products responsible for any observed effects.

## Results

### Untreated Samples

#### Proliferation

**Figure 1:**
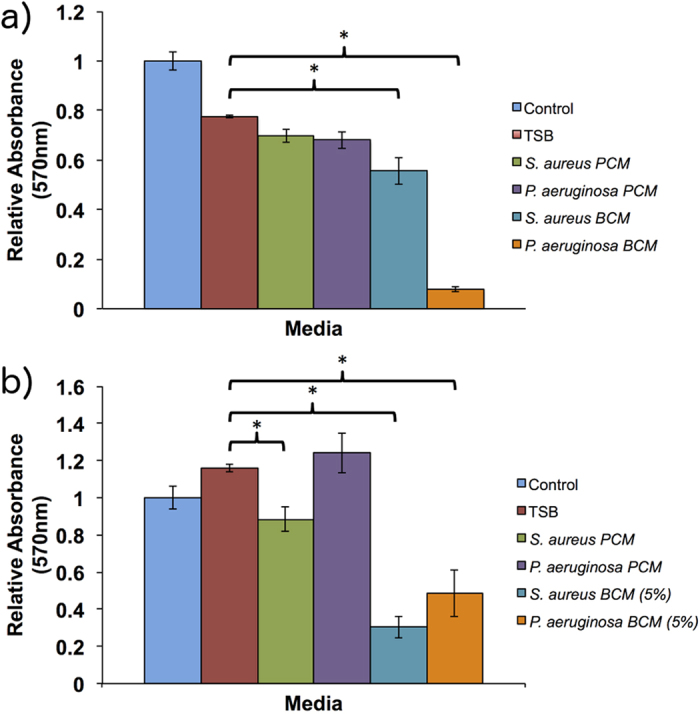
Effect of conditioned media on cell proliferation. BCM and PCM were tested for their effect on cell proliferation using hTMks (**a**) and hEKs (**b**). For hEKs the BCM was diluted further to 5% due to high levels of cell death. Additionally, compared to the TSB media control, PCM from *S. aureus* significantly reduced proliferation (p = 0.0025) in hEKs, but PCM from *P. aeruginosa* did not (p = 0.7). However, the BCM of both bacterial species significantly reduced proliferation (indicated by *) in both cell types. Error bars are standard deviation of the mean with n = 4. Control is cell culture media only.

A 10% dilution of biofilm conditioned media (BCM) and planktonic conditioned media (PCM) from *S. aureus* and *P. aeruginosa* were examined for their effect on cell proliferation *in vitro*. Included controls were a 10% dilution of tryptic soy broth (TSB) plus a positive control of cell culture media (DMEM+) only (Fig. 1). Compared to the TSB media control, PCM of both *P. aeruginosa* and *S. aureus* had no significant effect on cell proliferation in human tympanic membrane keratinocytes (hTMks) (Fig. 1a). In contrast, BCM from *P. aeruginosa* completely inhibited proliferation in hTMks with an absorbance reading equivalent to that of seeded cells with no proliferation period (data not shown). For *S. aureus* BCM, proliferation in hTMks was significantly reduced to approximately 71 ± 5.4% of the TSB media control (p ≪ 0.001).

In hEKs, (Fig. 1b) the BCM was diluted further to 5% due to high levels of cellular death seen with a 10% dilution. At 5% dilution, the PCM of *S. aureus* produced a significant decrease in proliferation (p = 0.0025) to 76% of the TSB control while the PCM from *P. aeruginosa* showed no significant effect (p = 0.7). Additionally, the BCM from *S. aureus* also significantly reduced proliferation to 30.3 ± 5.7% of the TSB control (p  0.01), while BCM from *P. aeruginosa* significantly (p = 0.003) reduced proliferation to 48.3 ± 12.4% of the TSB control.

#### Migration

**Figure 2:**
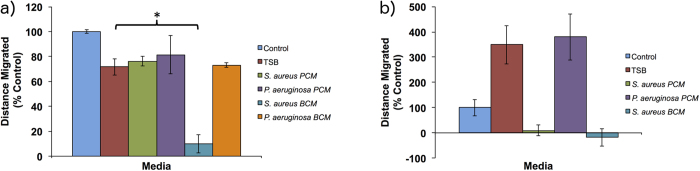
Effect of conditioned media on cell migration. In hTM keratinocytes (**a**) the PCM of both bacterial species in addition to the BCM of *P. aeruginosa* had no significant effect on migration. The BCM of *S. aureus* significantly reduced cell migration to 10% of the TSB control and 13% of the no media control. In hEKs (**b**), the TSM control in addition to *P. aeruginosa* PCM showed increased migration to over fold of cell culture media only. *S. aureus* PCM and BCM both seemed to prevent migration while *P. aeruginosa* BCM appeared toxic. Statistical significance (p < 0.05) is shown by *. Error bars show standard deviation of the mean with n = 4. Scale bars = 200 μm. Control is cell culture media only.

The effect of diluted PCM and BCM on cell migration was examined (Fig. 2). In hTMks, PCM from *S. aureus* and *P. aeruginosa* plus BCM from *P. aeruginosa* had no significant effect on migration compared to the TSB media control (p = 0.27, 0.29 and 0.72 respectively) (Fig. 2a). However, the BCM of *S. aureus* significantly reduced migration to 9.8 ± 7.2% of the media-only control and approximately 13% of the TSB media control (p ≪ 0.01) in this cell type.

In hEKs (Fig. 2b), the TSB media and PCM from *P. aeruginosa* seemed to accelerate migration to over 300% of the media-only control. However, the BCM from *P. aeruginosa* appeared to be highly cytotoxic.

**Figure 3:**
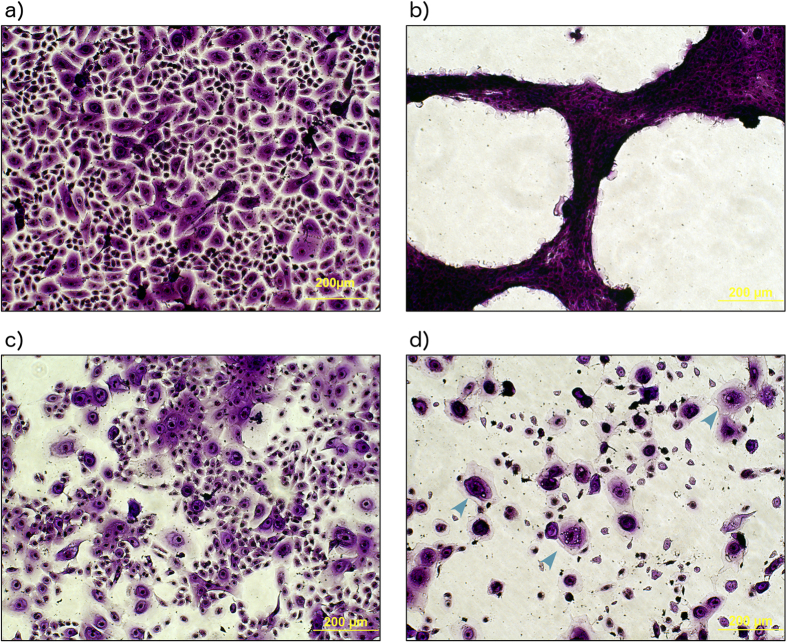
Effect of conditioned media on cell morphology. Stained with crystal violet, cells in the control group possessed a characteristic cobblestone appearance (**a**). *P. aeruginosa* BCM appeared to have a highly toxic effect with cells appearing to shrink and clump together (**b**). *S. aureus* PCM appeared to have a mild to moderate toxic effect and reduced cell numbers (**c**). However, the BCM of *S. aureus* also appeared highly toxic resulting in the presence of apoptotic cells (indicated by blue arrows) (**d**).

Under microscopy, control hEKs stained with crystal violet exhibited the characteristic cobblestone appearance of keratinocytes (Fig. 3a). When treated in 5% *P. aeruginosa* BCM for the duration of the migration experiment (15 hours) a large proportion of the cells were killed and appeared contracted (Fig. 3b). The PCM of *S. aureus* (Fig. 3c) resulted in a reduced number of cells with altered morphology, which appeared to be losing close contact with their neighbours. Treatment with BCM of *S. aureus* resulted in highly reduced cell numbers (Fig. 3d). In addition, the presence of apoptotic cells appeared to be evident (Fig. 3d, arrow heads). It was therefore not possible to interpret these results in terms of altered migration. However, the observations indicate that hEKs are more sensitive to conditioned media than hTMks.

### Effect of Heat Treatment

Only hTM-derived keratinocytes were used for analyses.

#### Proliferation

**Figure 4:**
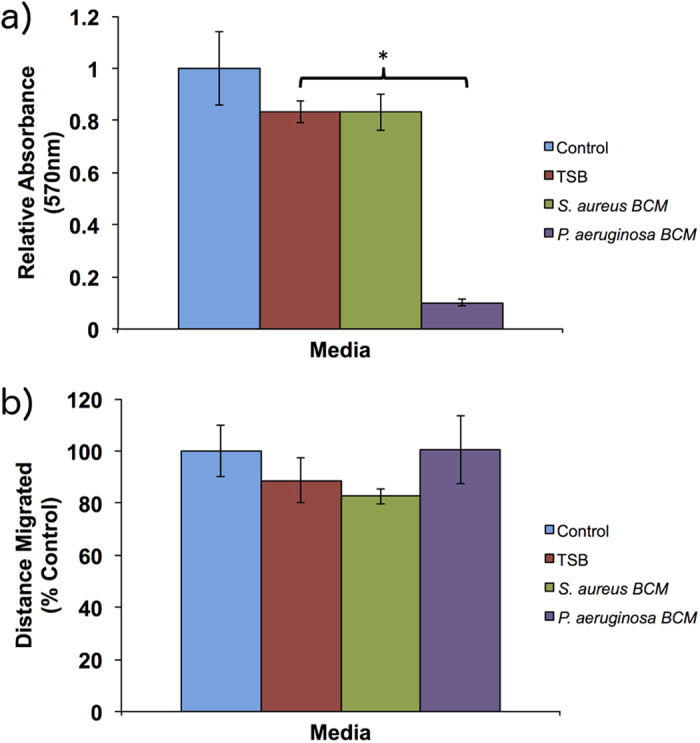
Effect of heat treated BCM on cell migration and proliferation. Heat treatment completely removed the anti-proliferative effect of *S. aureus* but had no effect on *P. aeruginosa* BCM, which significantly reduced proliferation (**a**). For migration (**b**) heat treatment also removed the inhibitory activity of *S. aureus* BCM. P < 0.05 * and error bars are the standard deviation of the mean with n = 4. Control is cell culture media only.

As the PCM has no observable effect on hTMks proliferation, it was excluded from this experiment. Heat treatment completely abrogated all anti-proliferative activity of *S. aureus* BCM, which showed no significant difference to the TSB media control (p = 0.97). However, heat treatment had no effect on the anti-proliferative activity of *P. aeruginosa* BCM (Fig. 4a).

#### Migration

Similar to proliferation, heat treatment of *S. aureus* BCM removed the ability to inhibit migration (Fig. 4b), which showed no significant difference to the TSB control (p = 0.23). Heat treated BCM from *P. aeruginosa* also demonstrated no significant difference compared to TSB control (p = 0.19), similar to its untreated form.

### Effect of Filtration

#### Proliferation

**Figure 5:**
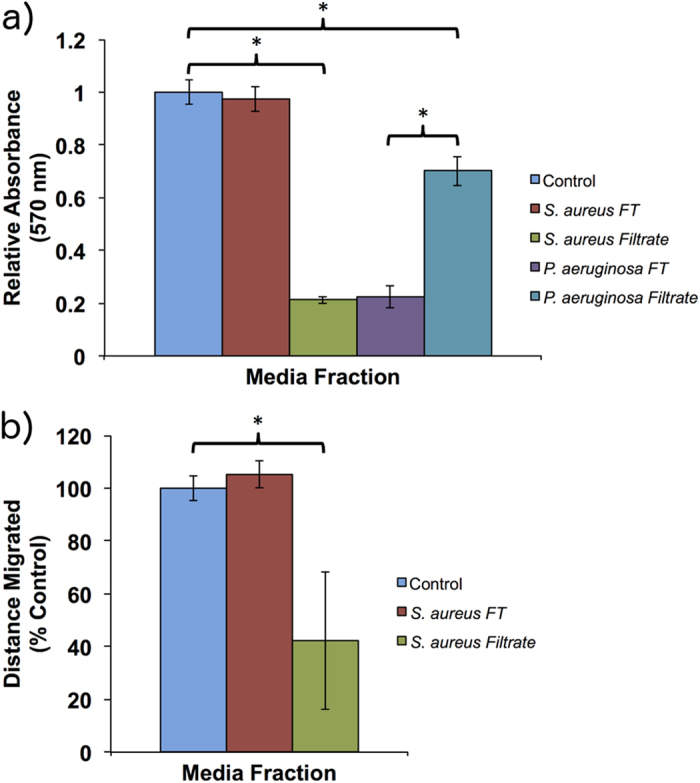
Effect of micro-filtered BCM on cell migration and proliferation. For proliferation (**a**) the effect of *S. aureus* were confined to the filtrate. However, the majority of activity for *P. aeruginosa* was seen in the flow through (FT) fraction. The migration assay was performed only on *S. aureus* BCM (**b**) and not *P. aeruginosa* due to the lack of activity seen in that species. All the inhibitory activity was seen in the filtrate fraction. Error bars are standard deviation of the mean with n = 4. Control is cell culture media only.

Fractionating the samples using a 3 KDa filter showed all the anti-proliferative activity of *S. aureus* was found in the filtrate (p ≪ 0.01), with no significant (p = 0.45) activity found in the flow through fraction (Fig. 5a). By contrast, *P. aeruginosa* activity was primarily in the flow through fraction with significant activity (p = 0.002) also detected in the filtrate. However, this activity was significantly less than that of the flow through (p ≪ 0.01).

#### Migration

Only BCM from *S. aureus* was tested (Fig. 5b) due to lack of activity in *P. aeruginosa* conditioned media. Similar to the effects on proliferation, the inhibitory effect of *S. aureus* on cellular migration was found only in the filtrate, which was significantly reduced to 40 ± 26.1% of the TSB control (p = 0.019). No significant activity (p = 0.255) was recorded for the flow through fraction.

### PAGE and Proteomics

**Figure 6:**
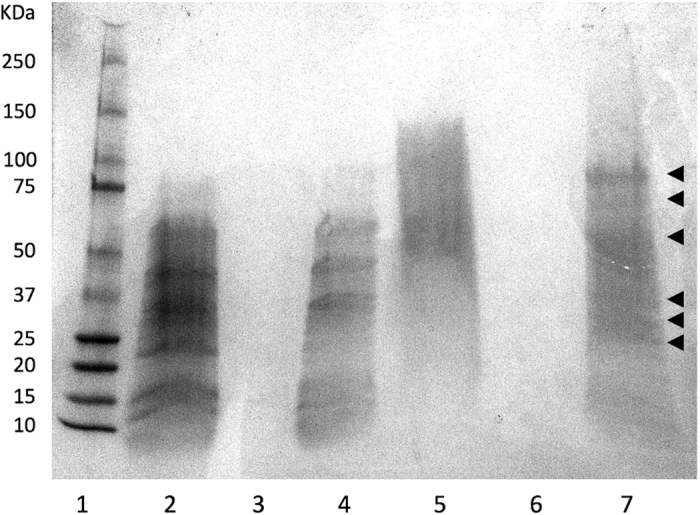
Page separation of proteins. Raw samples of BCM plus the microfiltered fractions of BCM were run on a 4–12% gradient polyacrylamide gel and stained with coomassie blue. Lane 1 = molecular size marker, 2 = *P. aeruginosa* raw BCM, 3 = *P. aeruginosa* flow through fraction, 4 = *P. aeruginosa* filtrate fraction, 5 = *S. aureus* raw BCM, 6 = *S. aureus* flow through fraction, 7 = *S. aureus* filtrate fraction. The arrows indicate bands that were extracted for peptide/protein identification.

PAGE analysis was performed on the BCM and PCM for both bacterial species. No protein bands were detectable in the TSB media control or PCM (gel not shown). In the BCM gel (Fig. 6), raw BCM from *P. aeruginosa* and *S. aureus* (Lanes 2 and 5 respectively) produced several discernable bands ranging in size from approximately 12 KDa to 80 KDa. Samples of flow through following fractionation were also included (lane 3 and 6) along with samples of the filtrate (Lane 4 and 7) for *P. aeruginosa* and *S. aureus* respectively. All proteins were retained in the filtrate. Six identifiable bands from *S. aureus* (arrows) were removed from the gel for peptide sequencing and identification. These are summarized in Table 1. Identification of proteins from *P. aeruginosa* BCM was not performed due to the inability of heat treatment to abrogate its effect and lack of activity in the protein fraction. These results suggest that activity was not due to a protein.

## Discussion

In this study, conditioned media derived from *P. aeruginosa* and *S. aureus* cultured as biofilms were examined for their ability to disrupt the normal wound healing processes of cell proliferation and migration. Keratinocytes from the tympanic membrane (TM) and the normal skin epidermis from the upper arm were chosen for the *in vitro* analysis due to their different healing patterns. When the dermis heals in response to injury, it undergoes sequential phases of haemostasis, inflammation, cellular proliferation, and finally cellular migration. In TM wound healing, the first two stages remain, but migration precedes proliferation^10^.

For hTMks, at the 10% dilution tested, BCM from *S. aureus* was capable of a modest reduction in proliferation, while it completely inhibited cellular migration. Conversely, the BCM of *P. aeruginosa* appeared to only affect proliferation with no significant effect on migration. However, the PCM from both species had no significant effect on either proliferation or migration. The hEKs appeared to be far more sensitive to the conditioned media. At 10% dilution, BCM from both species was highly toxic, effectively killing the hEKs in both migration and proliferation tests. However, when reduced to 5% dilutions a clear inhibitory effect on proliferation was seen. The BCM of both species significantly reduced the level of proliferation compared to the TSB control, with *S. aureus* BCM appearing to have a larger effect than that of *P. aeruginosa*. However, a comparison between *S. aureus* and *P. aeruginosa* BCM was found to be not significant (p = 0.083). In addition, the PCM of *S. aureus* also caused a significant decrease in proliferation, but was much less potent than the BCM. On the other hand, migration was more difficult to interpret. Both the PCM and BCM of *S. aureus* had a toxic effect on hEKs in addition to the BCM of *P. aeruginosa* (Fig. 3a–d). Therefore, meaningful migration results could not be firmly established. However, it should be noted that the PCM of *P. aeruginosa* had no significant effect compared to the TSB control, although both seemed to increase the rate of migration compared to the cell culture media only control.

Previous studies using cultured cells have examined the effects of PCM and BCM derived from *S. aureus.* Using wound scratch assays, both were found to significantly reduce gap closure in keratinocytes and fibroblast cells^11^,^12^. However, a difference in cell morphology was noted between PCM and BCM treated cells indicating potentially different mechanisms of action. Similarly, both PCM and BCM resulted in reduced viability in both cell types with the BCM being significantly more potent than PCM, which was also reflected in our studies using hEKs, but this did not seem the case in hTMks. For apoptosis, BCM and PCM possessed equal potency in fibroblasts^12^. However, in keratinocytes, BCM treatment caused significantly higher levels of apoptosis than PCM^11^, which was also suggested in our experiments. In the case of *P. aeruginosa*, much exists in the literature regarding the presence of biofilm in wounds and their effects. However, no similar *in vitro* studies into effects on proliferation and migration have been performed.

In an effort to determine which components of the BCM were exerting the effects seen, further analyses were performed on hTMks. Heat treatment and microfiltration on the BCM revealed the most likely cause for the effects seen for *S. aureus* was a secreted protein. This was based on heat treatment completely abrogating all *S. aureus* BCM effects, presumably due to denaturation. Similarly, after filtering the BCM with a 3 KDa cut-off, the majority of activity was preserved in the filtrate and not the flow through, which is suggestive of a retained, and relatively large molecular weight protein and not a small molecule. On the other hand, the effects caused by *P. aeruginosa* BCM appeared to be caused by a small molecule due to the ineffectiveness of heat treatment and the anti-proliferative effect being seen within the flow through fraction following microfiltration.

To further establish possible bioactive components of the conditioned media, SDS PAGE was performed. This revealed a complex pattern of secreted proteins for both *P. aeruginosa* and *S. aureus* BCM. As the data suggested only *S. aureus* activity was due to a protein(s), six of the more prominent *S. aureus* protein bands were excised from the gel and identified (Table 1). A number of identified proteins including transketolase, triosephosphate isomerase, purine nucleoside phosphorylase and glucosamine-6-phosphate deaminase are typically involved with essential metabolic processes with no known link to wound healing. However, of the proteins identified, several have been previously implicated in delayed wound healing and/or cell death and may be contributors to the effects seen in this study. These proteins include alpha hemolysin, a highly studied *S. aureus* cytotoxin that is particularly potent in lysing rabbit erythrocytes. In addition, it is also known to be dermonecrotic and neurotoxic^13^. Alcohol dehydrogenase (AD) may also require further study. Currently, no explicit reports on the direct effects of this enzyme with respect to wound healing have been reported. However, there exists anecdotal evidence that suggests the presence of AD may have a negative impact on wound healing. A transcriptome microarray study on healing tympanic membranes in rats revealed AD to be the most potently downregulated gene in the first 12 hours following an induced perforation^14^. Other studies have shown that ethanol exposure prior to injury may inhibit normal wound healing processes and have suggested the presence of ethanol itself plus its metabolites to be the likely cause^15^,^16^. However, the effects of elevated levels of AD were overlooked on these occasions. Therefore, combined with current data it is possible to consider that the presence of AD may also cause a negative impact on early wound healing processes. Fructose-bisphosphate aldolase (ALDOA) is a glycolytic enzyme and has been shown to directly bind to actin filaments of the cytoskeleton and modulate polymerization^17^. In keratinocytes, endogenous ALDOA is involved in forming actin based lamellipodia, which are structures important for cell migration^18^. Therefore, its plausible that exogenous ALDOA from *S. aureus* biofilm may alter the actin polymerisation process leading to a downstream effect in cell migration. Lactate dehydrogenase is an enzyme that reversibly converts lactate into pyruvate. In a healing environment, lactate accumulation stimulates the production of growth factors such as VEGF^19^ and TGF ß^20^ and is capable of enhancing cellular migration^21^. Similar to ALDOA, exogenous lactate dehydrogenase would effectively remove lactate from the wound environment and potentially reduce the pro-healing effects. Another interesting candidate identified was epidermal cell differentiation inhibitor (EDIN) protein. EDINs are able to ADP-ribosylate members of the Rho GTPase family causing their inactivation^22^. Inhibition of the Rho GTPase Rac1 results in disturbed migration of keratinocytes both *in vitro* and *in vivo*^23^. Additionally, EDIN produces a loss of barrier function in endothelial cells through actin cable disruption leading to the formation of microapertures. This provides direct access of the endothelium basement membrane to *S. aureus* bacterium^24^. Furthermore, EDIN genes from *S. aureus* strains have also been found to correlate to higher grade diabetic foot ulcers^25^. However, in this instance the presence of biofilm was not established.

In terms of wound healing there is the potential that biofilm secretions from different species may act in synergy. For example, there is a correlation between the *in vitro* biofilm - producing capacity of *P. aeruginosa* and *S. aureus* and unfavourable evolution after endoscopic sinus surgery^26^. In addition, using a porcine wound model, it was shown that while individual infections with strains of *S. aureus* and *P. aeruginosa* were sufficient to delay wound healing, only a co-infection resulted in decreased expression of KGF1^27^. In normal wound healing circumstances KGF1 acts through its receptor to increase migration and proliferation.

There were several limitations inherent in this study, primarily in controlling the many variables. For example, quantifying the amount of total protein present in each sample may have enhanced our results. However, this process was complicated by the presence of the trypsinised soy protein present in the bacterial culture media. Therefore, normalising for protein content was not possible. Similarly, controlling growth conditions of the planktonic and biofilm cultures were difficult to perform equally. This is due to the different growth rates and potential nutrient requirements for the different phases. Also, the process of freeze drying may result in the loss of compounds such as volatile organic chemicals. Additionally, it has also been noted that protein structure may be adversely affected^28^.

In this study we have shown that BCM from two of the most common bacterial strains associated with infections have a profound effect on the wound healing processes of migration and proliferation. Additionally, we have shown a differential effect in keratinocytes of different anatomical sites. Furthermore, the data suggests that effects caused by *P. aeruginosa* BCM were due to a small cytotoxic molecule while the effects from *S. aureus* BCM were protein based. Proteomics on the PAGE separated *S. aureus* BCM was able to reveal a potential cocktail of proteins that may act either individually or in synergy to produce the effects seen. Our data would suggest that in addition to identifying which species of pathogens are present in a chronic wound or infection, a more detailed study of biofilm components is also necessary to elucidate how the presence of biofilm may delay wound healing.

## Methods

### Bacterial Isolates

*P. aeruginosa* isolates used in this study were obtained from a clinical case of canine otitis externa from Australia. Swabs were cultured onto 5% Columbia sheep blood agar (SBA) (Thermo Fisher Scientific, Scoresby, Vic, Australia) and incubated at 37 °C and 42 °C, overnight. Following incubation, cultures were stained by the Gram-method for determination of purity and morphology and an oxidase test was performed. *S. aureus* ATCC 29213 was routinely streaked out onto SBA and incubated at 37°C overnight. All bacterial isolates were stored at −80 °C in brain heart infusion broth (BD Biosciences, Sydney, NSW, Australia) with 20% glycerol (Ajax Finechem (Thermo Fisher)).

### Conditioned Media

#### Planktonic and biofilm culture conditions

Inoculated tryptic soy broth (TSB) (25 ml culture concentration of 1.0 × 10^7 ^cfu/mL) was incubated in 50 ml tubes at 35 ± 2 ^°^C for 24 hours on a shaking incubator. Biofilms were formed using the MBEC™ high-throughput assay (MBEC™ Innovotech, Canada) according to the manufacturer’s instructions^29^ with incubation temperature at 35 ± 2 ^°^C and times extended to 24 hours. In brief, this device consists of a 96-peg-lid (top plate) and a ridged trough (bottom plate). The top plate fits over the trough containing 10 ml of TSB. The inoculated device is placed on a rocking platform (Select BioProducts, Model SBS300-2, New Jersey, United States), which allows a shear force to be created forming equivalent biofilms on all the pegs. The inoculum is incubated at 35 ± 2 ^°^C for 24 hours in the MBEC device before collection.

#### Supernatant preparation

Planktonic and/or biofilm cultures were centrifuged at 4500 rpm for 20 minutes at 4 °C (MPW-351R centrifuge, Warsaw, Poland), the pellet discarded, the supernatant filtered (Millex, PES 0.22 μm, Darmstadt, Germany) and frozen at −80 °C. Samples were freeze-dried (Dynavac Freeze Dryer, Model FD12, Australia) and stored at −20 °C. The freeze-dried conditioned media was then resuspended in an equivalent volume of DMEM+ (DMEM 4,500 mg/l D –glucose, supplemented with 100 U/ml penicillin, 100 g/ml streptomycin (Invitrogen (Thermo Fisher)), and 10% FBS (Invitrogen)) before further dilutions.

### Cell Culture

#### Ethical Statement

Isolation of human tympanic membrane keratinocytes was performed with approval from the St. John of God Healthcare Ethics Committee (Subiaco, Western Australia). Human normal skin keratinocytes were isolated from upper arm skin with approval from the University of Western Australian Human Research Ethics Committee (Approval # RA/4/1/5489). Patients signed a written, informed consent before intervention and specimens were treated anonymously. All collections and experiments were conducted according to the principles and ethical standards expressed by the Declaration of Helsinki.

#### Human Tympanic Membrane Keratinocytes (hTMk)

Culture of primary human tympanic keratinocytes was performed as previously described^30^. Briefly, fresh intact hTM tissue was obtained from consenting patients who underwent otological procedures requiring the permanent surgical removal of the tympanic membrane. All hTM tissue received was disease-free and considered morphologically normal. Small pieces of hTM tissue, approximately 0.25 mm^2^, were placed into BD Falcon 6-well culture plates (BD Biosciences) containing DMEM+ (Invitrogen). The derived primary cells were incubated for 20 days in a humidified cell culture incubator at 37 °C, with 5% carbon dioxide (CO_2_). Subsequent cell passages were incubated for 5 days in BD Falcon T-75 culture flasks (BD Biosciences).

#### Human Epidermal Keratinocytes (hEK)

Skin was excised from the upper arm and collected in 1 ml of Epigrow TM human epidermal keratinocyte complete media (Merck Millipore, Massachusetts, USA). Fatty tissue beneath the dermis was removed using a sterile surgical blade (No.23) and forceps. The skin tissue was then cut into 3–5 mm^2^ pieces. The pieces of skin were placed in 5 ml of EpigrowTM human epidermal keratinocyte complete media with 20 μl of fungizone (Gibco®, Invitrogen™, Carlsbad, California, USA) and 100 μl of kanamycin (Gibco®). Dispase (Life technologies™, Carlsbad, California, USA) was added at 0.0127 g per 5 ml (2.4 U/ml). This solution was incubated for 24 hours at 40 °C. This method is a modified version of a protocol published by the CELLNTEC company (CellNTEC, 2011).

After 24 hours, the epidermis was separated from the dermis using sterile fine tip forceps and the pieces of epidermis transferred to a sterile petri dish with 500 μl of TrypLE^TM^ Select (Gibco®). The epidermal tissue pieces were spread out flat on the surface of TrypLE^TM^ Select and covered with a lid to prevent evaporation. After incubating for 20 to 30 minutes at room temperature, the petri dish was tilted at a 30^0^ angle and 2 ml of human epidermal keratinocyte complete media was added. Using sterile forceps, epidermal tissue pieces were rubbed gently against the base of the petri dish in order to generate a single cell suspension. The resulting cell suspension was collected in a 15 ml centrifuge tube and the previous step repeated to increase the yield of keratinocytes. The second preparation of a single cell suspension was then added to the same 15 ml centrifuge tube. The combined cell preparation was then centrifuged at 200 g for 3 minutes. The cell pellet was resuspended in 5 ml of human epidermal keratinocyte complete media. Cells were cultured in a T25 tissue culture flask (Sarstedt Australia Pty Ltd, Belmore, NSW, Australia)

### Filtration

Samples of conditioned media were filtered through Amicon® Ultra-4 (Ultracel® - 3k) Centrifugal filters (Merk Millipore Ltd, Tullagreen, Cork, Ireland) with a 3 KDa cutoff. Both flow through and the filtrate were retained for analysis.

### Heat Treatment

Undiluted samples of conditioned media were subjected to heat treatment of 75 °C for 20 minutes. The samples were allowed to cool to room temperature prior to use in downstream assays.

### Proliferation Assay

Cell numbers following proliferation were calculated using a modified cell attachment assay^31^. This method uses a crystal violet solution to stain cells attached to the culture plate. After solubilizing the stained cells, the intensity of the solution is proportional to the number of cells present.

Cells were seeded into the wells of a 96 well plate (Greiner Bio-One GmbH, Frickenhausen, Germany) at a density of 3000 cells/well in 200 μl of DMEM+. The plates were incubated as described overnight (approx. 16 hours) to equilibrate. The media was then replaced with conditioned media diluted to 10% in DMEM+. Cells were incubated for 48 hours. The media was then removed and the cells fixed using 100 μl of 10% neutral buffered formalin (NBF). The fixed cells were washed twice in 100 μl PBS with gentle shaking. The PBS was aspirated and the cells stained with 100 μl of crystal violet solution (0.1% w/v crystal violet in 200 μM MES buffer, pH 6.0 filtered through a 0.22 μm filter) for 15 minutes. The crystal violet solution was removed and the cells washed 3 times in 100 μl ddH_2_0 with gentle shaking. All the ddH_2_O was aspirated and the cells solubilized in 100 μl of 10% acetic acid for 10 minutes with gentle shaking. Absorbance was read at 570 nm on an EPOCH microplate reader (BioTek Instruments Inc., Winooski, VT, USA)

### Migration Assay

Using IBIDI cell migration culture inserts (Ibidi GmbH, Martinsried, Germany), 5 × 10^4^ cells in a volume of 70 μL of DMEM+ were seeded into adjoining Ibidi insert chambers that had been placed into the wells of a 48-well plate (Greiner). The cells were then incubated overnight at 37 °C in a humidified atmosphere with 5% CO_2_. The inserts were removed after 24 hours to reveal a well defined gap approximately 500 μm wide. The wells were washed once by gentle shaking with 150 μl of PBS before adding 400 μl of fresh DMEM+ containing diluted conditioned media. Cells were photographed at 0 and 6 hours for hTMts and at 0 and 15 hours for hEKs using a Nikon Eclipse TE2000-5 microscope. Multiple photographs were taken to ensure the entire “gap” was captured. The gap was then measured using ImageJ software (NIH, USA) at 20 different points along the gap and averaged to provide the mean gap distance. The relative distance of cell migration is calculated as:

% Gap closure = Test (G-t_0_ hr – G-t_6/15_ hr)/Control (G-t_0_ hr – G-t_6/15_ hr) X 100%.

Where G-t_0_ hr is the mean gap distance prior to cellular migration immediately following the removal of the Ibidi culture insert (zero hour) and G-t_6/15_ hr is the mean gap distance at the 6 or 15 hour endpoint. The results are expressed as a percentage of the control treatment, which contains no conditioned media or diluted TSB media.

### Protein Analysis

#### PAGE

BCM and PCM samples (300 μl) were vacuum desiccated and resuspended in 20 μl of standard 1 × Laemmli loading buffer. Samples were heated to 95 °C for 10 minutes and allowed to cool to room temperature for 10 minutes. The tubes were centrifuged to collect the contents. Premade 4–12% gradient polyacrylamide gels were used for protein separation (Bio-Rad Laboratories, Hercules, CA, USA). The gel cassette was loaded into a Protean II gel apparatus (BioRad) as per the manufactures instructions with standard 1 × Tris/Glycine running buffer. The protein samples were loaded in the wells along with a sample of Kaleidoscope size marker (Promega Corporation, Sydney, Australia) and the gel run at 130 V for 30 minutes. After separation, the gel was removed from the cassette into a small dish and stained with 100 ml of coomassie blue stain (BioRad) for 2 hours with gentle rocking. The stain was decanted and the gel rinsed for several seconds in ddH_2_O to remove excess stain. 100 ml of destain solution (50% ddH_2_O, 40% methanol, 10% acetic acid) was then added to the gel along with 2–3 small pieces (2 cm^3^) of sponge and gently rocked overnight. The destained gel was then photographed.

#### Sequencing and Identification

Prominent bands in the polyacrylamide gel were excised and submitted to Proteomics International (Perth, Western Australia, Australia) for identification. Protein samples were trypsin digested and peptides extracted according to standard techniques^32^. Peptides were analysed by electrospray ionisation mass spectrometry using the Agilent 1260 Infinity HPLC system (Agilent Technologies, Santa Clara, CA, USA) coupled to an Agilent 6540 mass spectrometer (Agilent). Tryptic peptides were loaded onto a C18 column 300 SB, 5 μm (Agilent) and separated with a linear gradient of water/acetonitrile/0.1% formic acid (v/v). Spectra were analysed to identify proteins of interest using Mascot sequence matching software (Matrix Science Inc., Boston, MA, USA) with Ludwig NR database. A match was considered positive if two or more peptides map to the same protein and were within the predicted size parameters as estimated by the standard on the acrylamide gel.

### Statistics

ANOVA with a post hoc Dunnett’s test was used to analyse migration and proliferation results. Four replicates (n = 4) were used for each test. All comparisons were made against the TSB (vehicle) control. Significance was set at p < 0.05.

## Additional Information

**How to cite this article**: Jeffery Marano, R. *et al.* Secreted biofilm factors adversely affect cellular wound healing responses *in vitro*. *Sci. Rep.* **5**, 13296; doi: 10.1038/srep13296 (2015).

## Figures and Tables

**Table 1:** Identification of *S. aureus* proteins.

Band Number	Estimated Mass (KDa)	Sequence Matches	Mass (KDa)
1	80	Lipase 1	76714
		Bifunctional Autolysin	92280
2	75	Transketolase	72256
3	60	No sequence match	
4	35	Alpha-hemolysin	35953
		Alcohol dehydrogenase	36034
		Fructose-bisphosphate aldolase class 1	33007
		L-lactate dehydrogenase 1	34551
		Glycerophosphoryl diester phosphodiesterase	35289
		Chaperone hchA	32141
5	28	2,3-bisphosphoglycerate-dependent phosphoglycerate mutase	26663
		Triosephosphate isomerase	27303
		Epidermal cell differentiation inhibitor (EDIN)	27663
		Diacetyl reductase [(S)-acetoin forming]	27227
		Enterotoxin A	29656
6	25	Purine nucleoside phosphorylase	25892
		Glucosamine-6-phosphate deaminase	22330
		3-hexulose-6-phosphate synthase	22436

Bands excised from the PAGE gel were submitted for identification.

## References

[b1] Trostrup H. *et al.* Pseudomonas aeruginosa biofilm aggravates skin inflammatory response in BALB/c mice in a novel chronic wound model. Wound Repair Regen 21, 292–299 (2013). 23437978 10.1111/wrr.12016

[b2] Percival S. L. *et al.* A review of the scientific evidence for biofilms in wounds. Wound Repair Regen 20, 647–657 (2012). 22985037 10.1111/j.1524-475X.2012.00836.x

[b3] Wang A. Y. *et al.* Animal models of chronic tympanic membrane perforation: a ‘time-out’ to review evidence and standardize design. Int J Pediatr Otorhinolaryngol 78, 2048–2055 (2014). 25455522 10.1016/j.ijporl.2014.10.007

[b4] Cooper R. A., Bjarnsholt T. & Alhede M. Biofilms in wounds: a review of present knowledge. J Wound Care 23, 570–582 (2014). 25375405 10.12968/jowc.2014.23.11.570

[b5] Costerton J. W. Introduction to biofilm. Int J Antimicrob Agents 11, 217–21; discussion 237-9 (1999). 10394973 10.1016/s0924-8579(99)00018-7

[b6] Dotsch A. *et al.* The Pseudomonas aeruginosa transcriptome in planktonic cultures and static biofilms using RNA sequencing. PLoS One 7, e31092 (2012). 22319605 10.1371/journal.pone.0031092 PMC3272035

[b7] Sauer K., Camper A. K., Ehrlich G. D., Costerton J. W. & Davies D. G. Pseudomonas aeruginosa displays multiple phenotypes during development as a biofilm. J Bacteriol 184, 1140–1154 (2002). 11807075 10.1128/jb.184.4.1140-1154.2002 PMC134825

[b8] Bjarnsholt T. The role of bacterial biofilms in chronic infections. APMIS Suppl 1–58 (2013). 10.1111/apm.12099 23635385

[b9] Zhao G. *et al.* Delayed wound healing in diabetic (db/db) mice with Pseudomonas aeruginosa biofilm challenge: a model for the study of chronic wounds. Wound Repair Regen 18, 467–477 (2010). 20731798 10.1111/j.1524-475X.2010.00608.x PMC2939909

[b10] Gladstone H. B., Jackler R. K. & Varav K. Tympanic membrane wound healing. An overview. Otolaryngol Clin North Am 28, 913–932 (1995). 8559580

[b11] Kirker K. R. *et al.* Loss of viability and induction of apoptosis in human keratinocytes exposed to Staphylococcus aureus biofilms *in vitro*. Wound Repair Regen 17, 690–699 (2009). 19671124 10.1111/j.1524-475X.2009.00523.x PMC2749089

[b12] Kirker K. R., James G. A., Fleckman P., Olerud J. E. & Stewart P. S. Differential effects of planktonic and biofilm MRSA on human fibroblasts. Wound Repair Regen 20, 253–261 (2012). 22332802 10.1111/j.1524-475X.2012.00769.x PMC3292663

[b13] Dinges M. M., Orwin P. M. & Schlievert P. M. Exotoxins of Staphylococcus aureus. Clin Microbiol Rev 13, 16–34, table of contents (2000). 10627489 10.1128/cmr.13.1.16-34.2000 PMC88931

[b14] Santa Maria P. L., Redmond S. L., McInnes R. L., Atlas M. D. & Ghassemifar R. Tympanic membrane wound healing in rats assessed by transcriptome profiling. Laryngoscope 121, 2199–2213 (2011). 21919009 10.1002/lary.22150

[b15] Radek K. A., Kovacs E. J., Gallo R. L. & DiPietro L. A. Acute ethanol exposure disrupts VEGF receptor cell signaling in endothelial cells. Am J Physiol Heart Circ Physiol 295, H174–84 (2008). 18469146 10.1152/ajpheart.00699.2007 PMC2494747

[b16] Ranzer M. J., Chen L. & DiPietro L. A. Fibroblast function and wound breaking strength is impaired by acute ethanol intoxication. Alcohol Clin Exp Res 35, 83–90 (2011). 20958333 10.1111/j.1530-0277.2010.01324.x PMC3005009

[b17] Kusakabe T., Motoki K. & Hori K. Mode of interactions of human aldolase isozymes with cytoskeletons. Arch Biochem Biophys 344, 184–193 (1997). 9244396 10.1006/abbi.1997.0204

[b18] Tochio T., Tanaka H., Nakata S. & Hosoya H. Fructose-1,6-bisphosphate aldolase A is involved in HaCaT cell migration by inducing lamellipodia formation. J Dermatol Sci 58, 123–129 (2010). 20362419 10.1016/j.jdermsci.2010.02.012

[b19] Constant J. S. *et al.* Lactate elicits vascular endothelial growth factor from macrophages: a possible alternative to hypoxia. Wound Repair Regen 8, 353–360 (2000). 11115148 10.1111/j.1524-475x.2000.00353.x

[b20] Trabold O. *et al.* Lactate and oxygen constitute a fundamental regulatory mechanism in wound healing. Wound Repair Regen 11, 504–509 (2003). 14617293 10.1046/j.1524-475x.2003.11621.x

[b21] Beckert S. *et al.* Lactate stimulates endothelial cell migration. Wound Repair Regen 14, 321–324 (2006). 16808811 10.1111/j.1743-6109.2006.00127.x

[b22] Sugai M. *et al.* Epidermal cell differentiation inhibitor ADP-ribosylates small GTP-binding proteins and induces hyperplasia of epidermis. J Biol Chem 267, 2600–2604 (1992). 1733958

[b23] Tscharntke M. *et al.* Impaired epidermal wound healing *in vivo* upon inhibition or deletion of Rac1. J Cell Sci 120, 1480–1490 (2007). 17389689 10.1242/jcs.03426

[b24] Boyer L. *et al.* Induction of transient macroapertures in endothelial cells through RhoA inhibition by Staphylococcus aureus factors. J Cell Biol 173, 809–819 (2006). 16754962 10.1083/jcb.200509009 PMC2063895

[b25] Messad N. *et al.* Distribution of edin in Staphylococcus aureus isolated from diabetic foot ulcers. Clin Microbiol Infect 19, 875–880 (2013). 23176291 10.1111/1469-0691.12084

[b26] Bendouah Z., Barbeau J., Hamad W. A. & Desrosiers M. Biofilm formation by Staphylococcus aureus and Pseudomonas aeruginosa is associated with an unfavorable evolution after surgery for chronic sinusitis and nasal polyposis. Otolaryngol Head Neck Surg 134, 991–996 (2006). 16730544 10.1016/j.otohns.2006.03.001

[b27] Pastar I. *et al.* Interactions of methicillin resistant Staphylococcus aureus USA300 and Pseudomonas aeruginosa in polymicrobial wound infection. PLoS One 8, e56846 (2013). 23451098 10.1371/journal.pone.0056846 PMC3579943

[b28] Roy I. & Gupta M. N. Freeze-drying of proteins: some emerging concerns. Biotechnol Appl Biochem 39, 165–177 (2004). 15032737 10.1042/BA20030133

[b29] Inovotech. Instructions: The MBECTM High-throughput (HTP) Assay. **Revision 1**, http://www.innovotech.ca/MBEC_HTPInstructions_Rev1.pdf.

[b30] Redmond S. L., Levin B., Heel K. A., Atlas M. D. & Marano R. J. Phenotypic and genotypic profile of human tympanic membrane derived cultured cells. J Mol Histol 42, 15–25 (2011). 21072681 10.1007/s10735-010-9303-5

[b31] Humphries M. J. Cell-substrate adhesion assays. Curr Protoc Cell Biol Chapter 9, Unit 9.1 (2001). 10.1002/0471143030.cb0901s00 18228391

[b32] Bringans S. *et al.* Proteomic analysis of the venom of Heterometrus longimanus (Asian black scorpion). Proteomics 8, 1081–1096 (2008). 18246572 10.1002/pmic.200700948

